# In vitro Adipocyte Differentiation Inhibition and in vivo Effects on Lipid Metabolism in High-Fat Diet-Induced Obesity of *Euphorbia humifusa*

**DOI:** 10.4014/jmb.2308.08004

**Published:** 2023-11-10

**Authors:** Sung-Gyu Lee, Hyun Kang

**Affiliations:** Department of Medical Laboratory Science, College of Health Science, Dankook University, Cheonan 31116, Republic of Korea

**Keywords:** 3T3-L1, obesity, adipocyte differentiation, *Euphorbia humifusa*, SREBP-1c

## Abstract

*Euphorbia humifusa* Willd (*Euphorbiaceae*) is a functional raw material with various pharmacological activities. This study aimed to validate the inhibitory effect of *Euphorbia humifusa* extract (EHE) on adipocyte differentiation in vitro and in a high-fat-diet (HFD)-induced mouse model to evaluate the *E.a humifusa* as a novel anti-obesity and lipid metabolism enhancer agent. EHE effects on obesity and lipid metabolism were assessed in HFD-induced obese mice after 4-week treatments. Results were compared among four treatment groups (*n* = 7/group): low fat diet (LFD), high fat diet (HFD), and HFD-induced obese mice treated with either 100 or 200 mg/kg/day EHE (EHE100 and EHE200, respectively). EHE (50 to 200 μg/ml) and quercetin (50 μg/ml) significantly reduced 3T3-L1 preadipocyte differentiation (*p* < 0.001), in a concentration-dependent manner. EHE affected lipid metabolism, as evidenced by changes in serum lipid components. The HFD-EHE100 and HFD-EHE200 groups exhibited significantly (*p* < 0.05) reduced triglycerides (TG, 97.50 ± 6.56 and 82.50 ± 13.20 mg/dL, respectively) and low-density lipoprotein-cholesterol (LDL-c: 40.25 ± 4.99 and 41.25 ± 6.36 mg/dL, respectively) compared to the HFD group (TG: 129.25 ± 19.81 mg/dL; LDL-c: 51.75 ± 11.59 mg/dL). Haematoxylin and Eosin (H&E) and Oil red O staining showed that EHE markedly reduced lipid accumulation and inhibited lipogenesis in the liver. Interestingly, EHE significantly (*p* < 0.01) reduced the expression of adipogenic transcription factors in liver tissue. Our results indicated that EHE has the potential to be a therapeutic agent for addressing obesity and lipid metabolism.

## Introduction

A World Health Organization (WHO) survey showed that obesity affects more than 300 million people worldwide, and it is not limited to developed countries. A recent study showed that more than 100 million people in developing countries are obese [1]. In addition to making life uncomfortable, obesity raises blood lipid levels, which leads to arteriosclerosis and heart disease. Obesity also increases insulin resistance, which leads to complications such as diabetes, irregular menstruation, and cancer. Thus, obesity induces chronic geriatric diseases, like hyperlipidemia, hypertension, coronary artery disease, and stroke. Therefore, obesity treatment and prevention are essential [2, 3].

Diet and exercise therapy are optional for treating obesity, but that treatment takes time and effort to show an effect. Consequently, demand has increased for treatment through supplements or drugs. Orlistat (Xenical) is an obesity treatment currently on the market. Orlistat inhibits gastric and pancreatic lipases in the lumen of the gastrointestinal tract to reduce systemic absorption of dietary fat. However, its use is restricted, due to side effects that cause fatty stools and bowel incontinence [4, 5]. Therefore, there is a need for functional raw materials derived from safe natural raw materials that can effectively control weight and avoid the side effects of artificially synthesized materials.

It has been suggested that oxidative stress is a critical link between obesity and its associated complications [6]. Various biochemical mechanisms might underlie the effects of obesity on systemic oxidative stress, such as NOX activation [7], glyceraldehyde auto-oxidation, oxidative phosphorylation, polyol and hexosamine pathways, PKC activation, hyperleptinemia [8], low antioxidant defense, chronic inflammation [9], and postprandial generation of reactive oxygen species [6].

Obesity can be characterized by increments in adipocyte size (hypertrophy), due to excessive lipid accumulation, and adipocyte number (hyperplasia), due to adipogenesis (*i.e.*, differentiation of preadipocytes into mature adipocytes). Various factors were found to have an influential impact on adipocyte hypertrophy and hyperplasia [10]. Adipogenesis is a multi-step process that involves a cascade of adipocyte-specific genes and transcription factors. Preadipocytes, represented by 3T3-L1 cells, differentiate into mature adipocytes in response to various hormones and adipogenic transcription factors. Inhibiting these transcription factors may be a key mechanism for suppressing fat accumulation [11].

*Euphorbia humifusa* Willd (*Euphorbiaceae*) is an annual plant that grows wild in eastern Asia. It grows to 20 cm in height, and it is generally glabrous, fine and slender, with multiple branched stems and opposing leaves [12]. The main chemical components of *E. humifusa* include flavonoids, garlic acid, and tannins. As pharmacological agents, these components have antioxidant and antibacterial activities against *Staphylococcus aureus*, diphtheria, *Escherichia coli*, and *Pseudomonas aeruginosa* [13]. In addition, *E. humifusa* is known to be effective against various types of cancer, inflammation, asthma, diabetes, heart disease, kidney disease, malignant headache, and mental anxiety, due to its excellent anticancer, detoxification, antibacterial, and sedative effects [14,15]. *E. humifusa* is known to contain various bioactive compounds such as alkaloids, flavonoids, terpenoids, and phenolic compounds, which possess physiological activities. These compounds are associated with anti-inflammatory, antioxidant, improvement of lipid metabolism, and anti-obesity properties. These attributes are believed to be relevant to the potential medicinal effects of *E. humifusa* and are particularly noteworthy. However, while research on anti-inflammatory and antioxidant properties is underway, studies regarding lipid metabolism and anti-obesity effects are lacking. Therefore, it is important to understand the potential health benefits of *E. humifusa* and establish a scientific basis for its traditional medicinal uses, given the current lack of research on aspects such as lipid metabolism and anti-obesity effects.

This study aimed to validate the potential inhibitory effect of *E. humifusa* extract (EHE) on adipocyte differentiation both in vitro using 3T3-L1 preadipocytes and in a high-fat diet (HFD)-induced mouse model. The objective was to evaluate *E. humifusa* as a novel anti-obesity agent by assessing its effects on obesity and lipid metabolism.

## Materials and Methods

### Preparation of *Euphorbia humifusa* Extract (EHE)

*Euphorbia humifusa* was purchased from Gyeong-dong market, Korea, in September 2019. It was taxonomically identified by a plant biotechnologist, Dr. Jong Bo Kim (College of Biotechnology, Konkuk University Glocal Campus, Republic of Korea). A voucher specimen was deposited at the College of Health Science, Dankook University, Korea. The dried stems and leaves of *E. humifusa* were crushed and extracted in a 10-fold volume of 70% ethanol for 72 h. The extract was filtered through filter paper (300-mm diameter circles, Advantech, Japan), then concentrated under reduced pressure at 55°C, and freeze-dried.

### DPPH Radical Measured with Electron Spin Resonance (ESR)

The radical scavenging effect of EHE on 2, 2-diphenyl-1-picrylhydrazyl (DPPH, Sigma-Aldrich, USA) was evaluated according to the method described previously by Lee *et al*. [16]. Briefly, 60 μl of EHE was added to 60 μl of 60 μM DPPH in methanol. After mixing strongly for 10 s, the mixture was moved into a capillary tube, and after 2 min, the scavenging of DPPH radicals was measured in each sample with an electron spin resonance (ESR) spectrometer (JES-FA, Jeol Ltd., Japan).

### High-Performance Liquid Chromatography (HPLC) Analysis

The composition of quercetin, an active component of EHE, was analyzed with high-performance liquid chromatography (HPLC), performed with a CBM-20A Communications Bus Module HPLC system equipped with an SPD-M20A diode array detector (Shimadzu, Japan). The analysis parameters are shown in Table 1.

### Cell Viability

To measure the cell viability of EHE, a 3-(3,4-dimethyl-thiazolyl-2)-2,5-diphenyl tetrazolium bromide (MTT) assay was performed, according to the method described previously by Carmichael *et al*. [17]. Briefly, cells were seeded into a 96-well plate (1 × 10^4^ cells/well). Then, EHE and quercetin were added at various concentrations in fresh medium. Absorbance was measured at 550 nm with an ELISA reader (Spectra MAX M2, Molecular Devices Inc, USA). Cytotoxicity was expressed as the percentage of absorbance from the test sample, compared to the absorbance of the control.

### 3T3-L1 Preadipocytes Culture

Adipocyte differentiation experiment was carried out by the method of Katja *et al*. [18] with a slight modification. The 3T3-L1 cells were purchased from ATCC (USA). Cells were cultured at 5% CO_2_ and 37°C in DMEM (Dulbeccós modified Eaglés medium)-high glucose (Invitrogen) supplemented with 10% bovine calf serum (BCS; Invitrogen, USA) and 1% antibiotics (Invitrogen). When the cells became confluent, they were seeded into 6-well plates (3 × 10^5^ cells/well) for 48 h. Then, 10% BCS, 23 mg/ml Isobutylmethylxanthine (IBMX)(Sigma-Aldrich, Chem. Co., USA), and 5 mg/ml insulin (Sigma-Aldrich) were added to medium supplemented with 1 mM dexamethasone (Sigma-Aldrich) to induce differentiation for 48 h. Then, every 2 days, the medium was replaced with DMEM medium supplemented with 5 mg/ml insulin and 10% fetal bovine serum. The EHE and quercetin treatments were added at the time the cells were placed in differentiation induction medium.

### Oil Red O Staining

After 3T3-L1 precursor adipocytes had differentiated for 9 days, the medium was removed. Next, the cells were fixed with 10% formaldehyde, and washed again with PBS. Then, Oil red O solution was added, and cells were stained at room temperature (RT) for 30 min. After removing the Oil red O solution, cells were washed with distilled water (DW), dried, and observed with a phase contrast microscope. Finally, the stained lipid droplets were dissolved in 100% isopropyl alcohol, and the absorbance was measured at 510 nm.

### Animal Experiments

For the in vivo study, five-week-old C57BL/6J (DBL Inc. Eumseong, Republic of Korea) male mice were purchased and acclimatized for one week. To induce obesity, mice were fed an HFD (60 kcal% Research Diet) for 4 weeks. The control group was fed a low-fat diet (LFD: 10 kcal% Research Diet, USA). The animals were divided into the following four treatment groups: LFD group (*n* = 7), HFD group (*n* = 7), HFD plus 100 mg/kg/day EHE (HFD-EHE100 group, *n* = 7), and HFD plus 200 mg/kg/day EHE (HFD-EHE200 group, *n* = 7). The kennel temperature was controlled at 21 ± 2°C and 50 ± 10% humidity with a 12-h light/dark cycle. After the completion of the experiment, the mice were euthanized using 5% isoflurane, and blood and tissue samples were collected. All animal experiments in this study were approved by the Dankook University Animal Experiment Committee (DKU-20-025).

### Measurement of Body Weight and Food Efficiency Ratio (FER)

During the 4-week experimental period, drinking water and food were provided freely, and body weight and food intake were measured at weekly intervals. The food efficiency ratio (FER) is calculated by dividing the weekly animal weight gain by the amount of food consumed.

FER (%) = (body weight gain (g)/food intake (g)) × 100

### Preparation of Serum and Tissue

After completing the experiment, the animals were fasted for 12 h before sacrifice, and blood was collected through the orbital vein. The serum was separated by centrifugation at 1,500 ×*g* for 15 min. After blood collection, the tissues of experimental animals were rinsed several times with PBS, and the surface moisture was removed. Finally, the tissues were weighed, then stored at −70°C.

### Biochemical Analysis of Serum

Aspartate aminotransferase (AST), alanine transaminase (ALT), and lipid content were analyzed with a biochemical analyzer (Konelab20XT, Thermo, USA). The atherosclerosis index (AI) was calculated, based on high-density lipoprotein-cholesterol (HDL-c) and total cholesterol (TC) contents, with the following formula:(TC-HDL-c)/HDL-c.

### Real-Time Polymerase Chain Reaction (RT-PCR)

Total RNA was extracted from abdominal fat tissues using TRIzol reagent (ThermoFisher Scientific). AccuPower RT PreMix (Bioneer, Inc., USA) and RNA-to-cDNA Kit (Bioneer, Inc., Republic of Korea) used properly according to the procedures provided by the manufacturer. The mRNA expression of targeted genes including Acyl-CoA synthetase-1 (ACS1), fatty acid synthesis (FAS), fatty acid transport-1 (FATP1), and Perilipin was measured using a thermal cycler (Takara Biotechnology Co., Ltd.). Each reaction was performed in the final volume of 20 μl including 1 μl of cDNA and 200 nM of each primer, with the thermocycle program consisting of an initial hot start cycle at 95°C for 30 s, followed by 40 cycles at 95°C for 5 s, 60°C for 15 s, and 72°C for 10 s. The dissolution curve started at 55°C and increased by 0.5°C to the end point of 95°C, reacting 80 times to detect the desired fluorescence value. Oligonucleotide sequences are presented in Table 2.

### Histological Analysis

Histological analyses were performed on liver and abdominal subcutaneous adipose tissue sections. Briefly, tissues were fixed in 10% formaldehyde solution for 24 h, then stained with H&E. Oil red O staining was performed to confirm the degree of fat accumulation in the liver cells. The stained slides were observed with an optical microscope (TS100, Nikon, Japan) at 200× magnification. Consecutive fat cells in the gonadal fat pad of mice were selected for size measurements. Adipocyte diameters were measured with ImageJ 20 software.

### Western Blot Analysis

Liver tissue was homogenized in RIPA buffer (with phosphatase and protease inhibitors). The liver tissue was extracted at 4°C for 30 min. Afterward, the supernatant was obtained by centrifugation at 18,928 ×*g* for 15 min. The protein concentration in the supernatant was determined using a protein analysis kit. 20 μg of cellular proteins were electrophoresed on a 10% sodium dodecyl sulfate-polyacrylamide gel (SDS-PAGE) and then transferred to an Immobilon-P membrane (Millipore, USA). The Western blot membrane was probed with specific antibodies (PPAR-γ, SREBP-1c, and C/EBP-α) and chemiluminescence (ECL) was used for detection.

### Statistical Analysis

Analyses were performed with Statistical Package for Social Science, v. 18.0 (SPSS Inc., USA). Statistical significance was evaluated with one-way analysis of variance (ANOVA), at the level of *p* < 0.05. Comparisons between groups were performed with Duncan's multiple range test.

## Results

### DPPH Radical Scavenging Effect

**Fig. 1:**
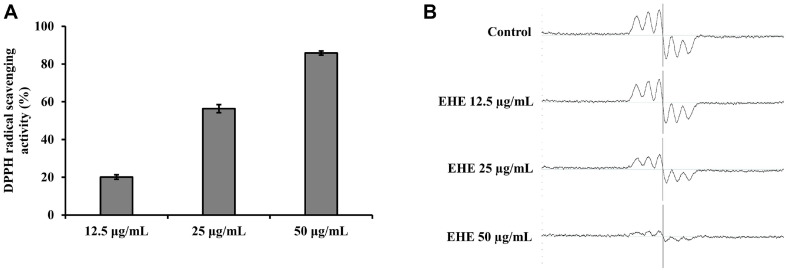
EHE scavenging activity on DPPH radicals. (**A**) The percentages of DPPH radicals scavenged at different EHE concentrations. (**B**) ESR spectrum of DPPH free radicals. All data are expressed as the mean ± SD of the experiment.

Fig. 1 shows the ESR results of EHE scavenging the DPPH radical. Fig. 1B shows the ESR spectrum of the DPPH free radical. The DPPH scavenging effect was calculated by taking the double integral of the ESR signal and quantifying it as a curved peak area. As the height of the peak decreased, the peak area decreased, which indicated an increase in the radical scavenging effect. The results confirmed that the EHE scavenging activity on DPPH radicals increased in a concentration-dependent manner (Fig. 1).

### Identification of Active Compound in EHE

**Fig. 2:**
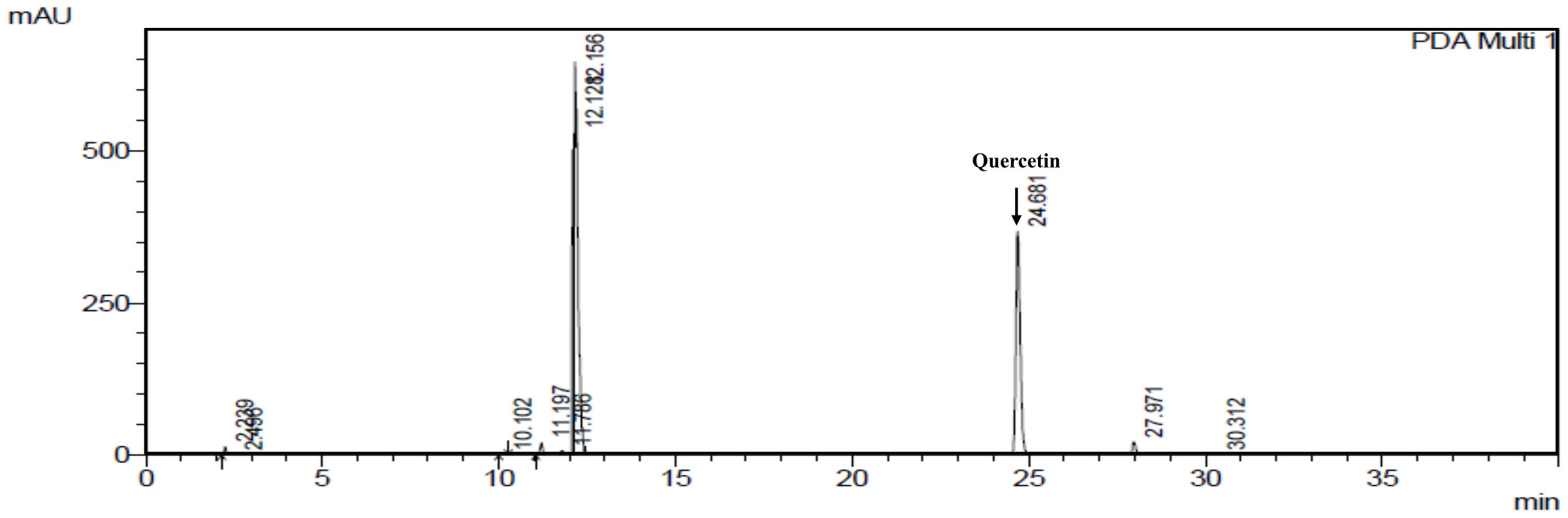
HPLC chromatogram shows quercetin content in EHE. Arrow indicates the quercetin peak.

The analytical HPLC chromatogram of the quercetin content in EHE is shown in Fig. 2. A calibration curve was established with a quercetin standard. The quercetin content in EHE was 17.098 mg/g.

### Effect of EHE on Adipocyte Viability and Lipid Accumulation

**Fig. 3:**
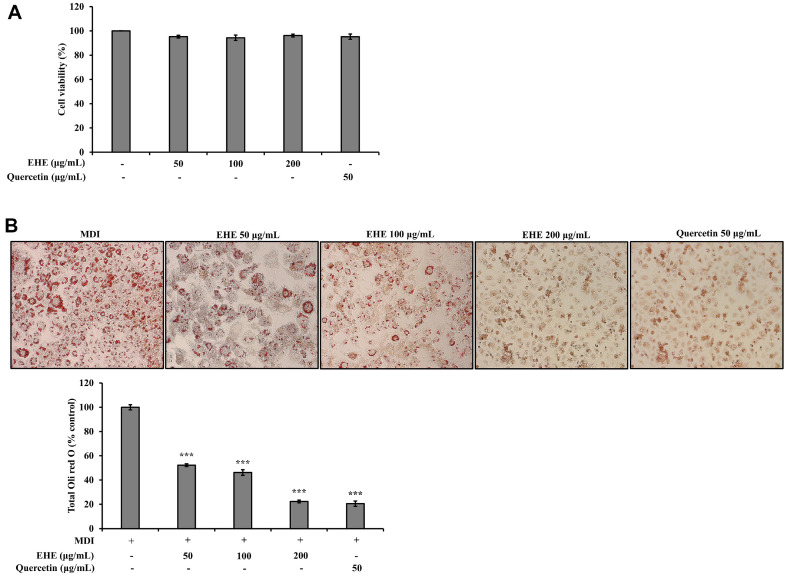
Effects of EHE and quercetin on the viability and differentiation of 3T3-L1 preadipocytes. (**A**) MTT assay results show cell viability after treatment with EHE (50, 100, and 200 μg/ml) and quercetin (50 μg/ml) for 24 h. (**B**) Images show Oil Red O stained cells (200 × magnification). Graph shows the quantification of intracellular lipids. Adipogenesis was induced with IBMX, dexamethasone, and insulin (MDI). EHE and quercetin treatments inhibited lipid accumulation. The data are presented as the mean percentage compared to control DMSO-treated cells (MDI). All data are expressed as the mean ± SD of the experiment. ***p < 0.001 compared to the MDI control group.

Prior to measuring the anti-adipogenic effects of EHE and quercetin, an MTT assay was performed to investigate dose-dependent cytotoxic effects in 3T3-L1 cells. Cells were treated with different concentrations of EHE (50, 100, and 200 μg/ml) or quercetin (50 μg/ml) for 24h. Compared to the control group, at all concentrations of EHE and quercetin, the survival rate was over 90% (Fig. 3A). Consequently, the maximum sample treatment concentration was set to 200 μg/ml. When preadipocytes differentiate during adipogenesis, intracellular triglyceride (TG) accumulation generates lipid droplets. A microscopic examination of cells grown in differentiation conditions showed that EHE significantly reduced the degree of fat accumulation (*p* < 0.001) compared to the control group (Fig. 3B).

### Changes in Body Weight, FER, and Organ Weight

**Fig. 4:**
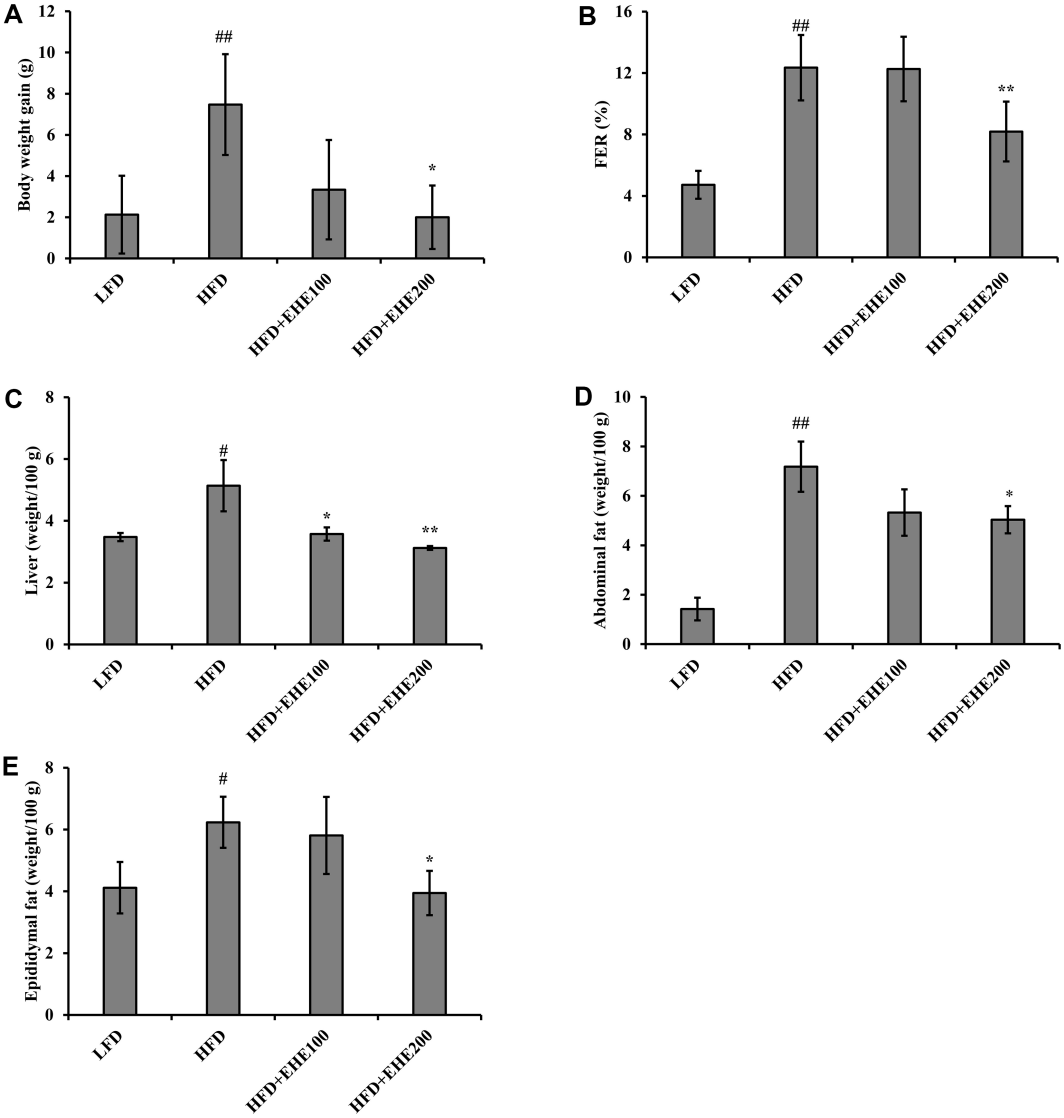
EHE regulation of body weight gain, FER, and tissue weights of HFD-fed mice. (**A**) Mean total body weight gains in each mouse group at the end of the 4-week treatment period. (**B**) FER. (C-E) Mean tissue weights per 100 g of tissue in each mouse group for (**C**) liver, (**D**) abdominal subcutaneous fat, and (**E**) epididymis fat. All values are expressed as the mean ± SD (*n* = 7). Different symbols indicate a significant difference (*p* < 0.05), based on Duncan’s multiple range test. #*p* < 0.05 and ##*p* < 0.01, compared to the LFD group; *p < 0.05 and **p < 0.01, compared to the HFD group.

To determine the effects of EHE on body and tissue weights in C57BL/6J HFD-induced obese mice, EHE was orally administered at 100 or 200 mg/kg, daily for 4 weeks. A negative control group was received DW. After 4 weeks, it was observed that the HFD group had a significant increase in body weight when compared to the LFD group. In contrast, the HFD+EHE200 group exhibited a significantly lower increase in body weight compared to the HFD group, resulting in a mean body weight gain similar to that observed in the LFD group (Fig. 4A). According to the results of FER measurements, the HFD group showed significantly higher FER compared to the LFD group (*p* < 0.01). Additionally, the FER of the group administered EHE at 200 mg/kg was significantly lower than that of the HFD group (*p* < 0.01) (Fig. 4B). Therefore, it is inferred that the treatment with EHE led to a reduction in body weight and food efficiency in the HFD group due to the pharmacological actions of EHE. To investigate the effect of EHE administration on individual tissues, liver, abdominal fat, and epididymal fat were excised, and the weights were measured. The HFD group gained significantly more weight in the liver, abdominal fat, and epididymal fat, compared to the LFD group. However, all tissue weights in the EHE-treated group were significantly reduced compared to the HFD group (Fig. 4C-4E). These results confirmed that EHE administration had both an anti-obesity effect and an inhibiting effect on HFD-induced hypertrophy of the liver and adipose tissues.

### Effect of EHE on Adipocyte Morphology and the Expression of Adipogenesis Genes in Abdominal Adipose Tissue

**Fig. 5:**
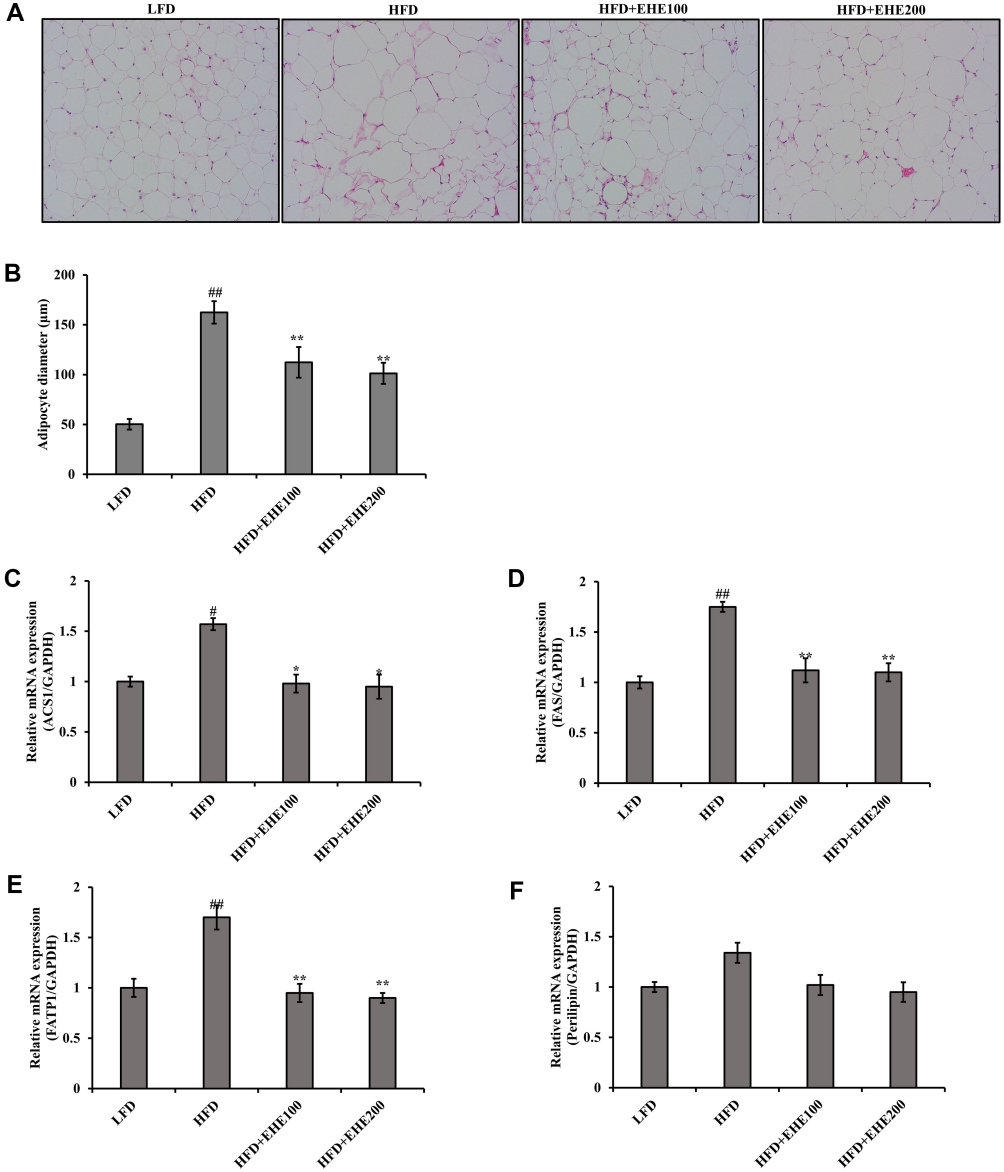
Effects of EHE on abdominal fat tissue morphology and expression of mRNA in the adipocyte differentiation-related genes in abdominal adipose tissues of HFD-fed mice. (**A**) Abdominal fat tissue histology. Representative adipose tissue sections stained with H&E (200× magnification) show adipocyte sizes. (**B**) Average diameter of adipocytes in adipose tissues from each group. Adipocyte RNA was isolated, and real-time PCR analysis was performed to determine mRNA expressions of (**C**) ACS1, (**D**) FAS, (**E**) FATP1, and (**F**) perilipin. GAPDH was used as an internal control. All data are expressed as the mean ± SD of the experiment. #*p* < 0.05 and ##*p* < 0.01 compared to the LFD group; *p < 0.05 and **p < 0.01 compared to the HFD group.

EHE had an effect on the morphology of abdominal adipose tissue in obese mice. Microscopic observations of abdominal fat cells showed that the size and arrangement of fat spheres were regular in the LFD group. On the other hand, abdominal fat cells in the HFD group showed severe fat deposition, and very large fat globules. However, the size of abdominal fat cells did not increase significantly in the EHE-treated group, compared to the HFD group (Fig. 5A and 5B).

The ACS1, FAS, and FATP1 genes are important for adipocyte differentiation. The mRNA expression levels of ACS1 increased considerably in the HFD group, compared with the LFD group, but were significantly decreased in the HFD+EHE groups, compared with the HFD group (*p* < 0.05) (Fig. 5C). mRNA expression of FAS and FATP1 gene was significantly higher in the HFD group than in the LFD group, but showed a significant decrease in the HFD+EHE groups, compared with the HFD group (*p* < 0.01) (Fig. 5D and 5E). The mRNA expression levels of Perilipin gene in all experimental groups was not significant, (Fig. 5F). The mRNA expression levels of ACS1, FAS, and FATP1 genes in adipose tissue in the HFD+EHE groups were significantly lower than those in the HFD group.

### Effect of EHE on Serum Lipid Levels, AST, and ALT Concentrations

**Fig. 6:**
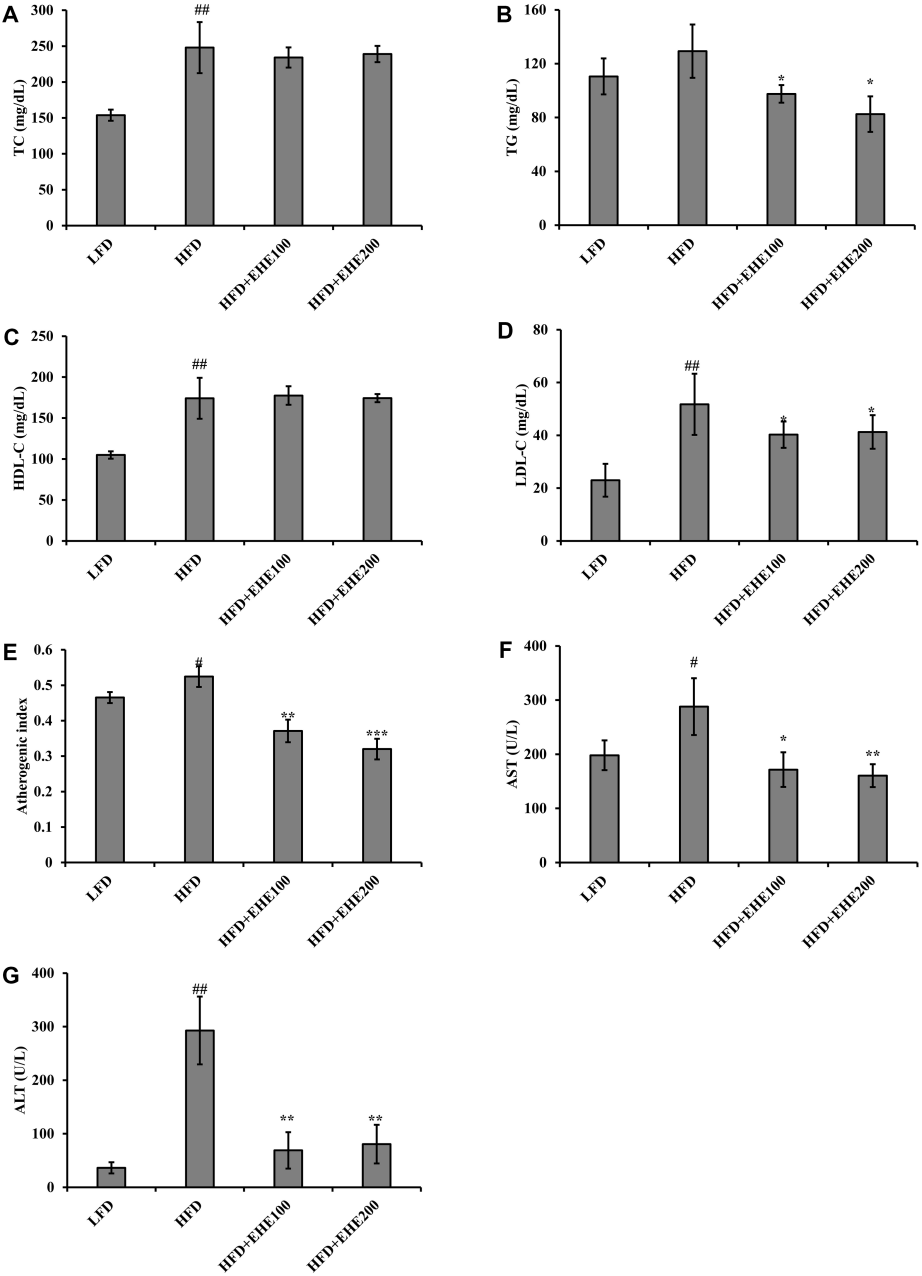
Effects of EHE on lipid levels, AST, and ALT in serum of HFD-fed mice. Serum concentrations of (**A**) TC, (**B**) TG, (**C**) HDL-c, (**E**) LDL-c, (**E**) the atherogenic index, (**F**) AST, and (**G**) ALT are expressed as the mean ± SD (*n* = 7). The atherogenic index was calculated with the following formula: (TC-HDL-c)/HDL-c. #*p* < 0.05 and ##*p* < 0.01 compared to the LFD group; *p < 0.05, **p < 0.01, and ***p < 0.001 compared to the HFD group.

The effects of EHE on serum lipid levels are shown in Fig. 6. In the LFD group, the serum levels of TC, TG, HDL-c, and low-density lipoprotein-cholesterol (LDL-c) were 153.75 ± 7.76, 110.50 ± 13.40, 104.90 ± 4.52, and 23.00 ± 6.21 mg/dL, respectively. In comparison, the mice fed an HFD for 8 weeks showed a significant (*p* < 0.01) increase in serum lipid content, with levels of 248.00 ± 35.59, 129.25 ± 19.80, 174.25 ± 25.08, and 51.75 ± 11.58 mg/dL, respectively. In contrast, the HFD + EHE100 and HFD + EHE200 groups showed significantly lower TG levels (97.50 ± 6.55 and 82.50 ± 13.20 mg/dL, respectively, *p* < 0.05) and LDL-c levels (40.25 ± 4.99 and 41.25 ± 6.36 mg/dL, respectively; *p* < 0.05), compared to the HFD group. In addition, the AI was significantly increased (*p* < 0.05) in the HFD group (0.52 ± 0.03) compared to the LFD group (0.47 ± 0.02), but it was significantly reduced (*p* < 0.01) in the HFD + EHE100 (0.37 ± 0.03) and HFD + EHE200 (0.32 ± 0.03) groups (Fig. 6E).

Changes in serum AST and ALT enzyme activities were measured to examine whether EHE affected the degree of liver damage in mice fed an HFD (Fig. 6F and 6G). In the HFD group, the mean AST level increased significantly (*p* < 0.05) compared to the LFD group. In contrast, the HFD + EHE200 group showed a significantly lower AST level (*p* < 0.01) compared to the HFD group. Similarly, serum ALT increased by about 8-fold in the HFD group compared to the LFD group, but the levels were similar between the HFD + EHE groups and the LFD group.

### Effect of EHE on Liver Tissue

**Fig. 7:**
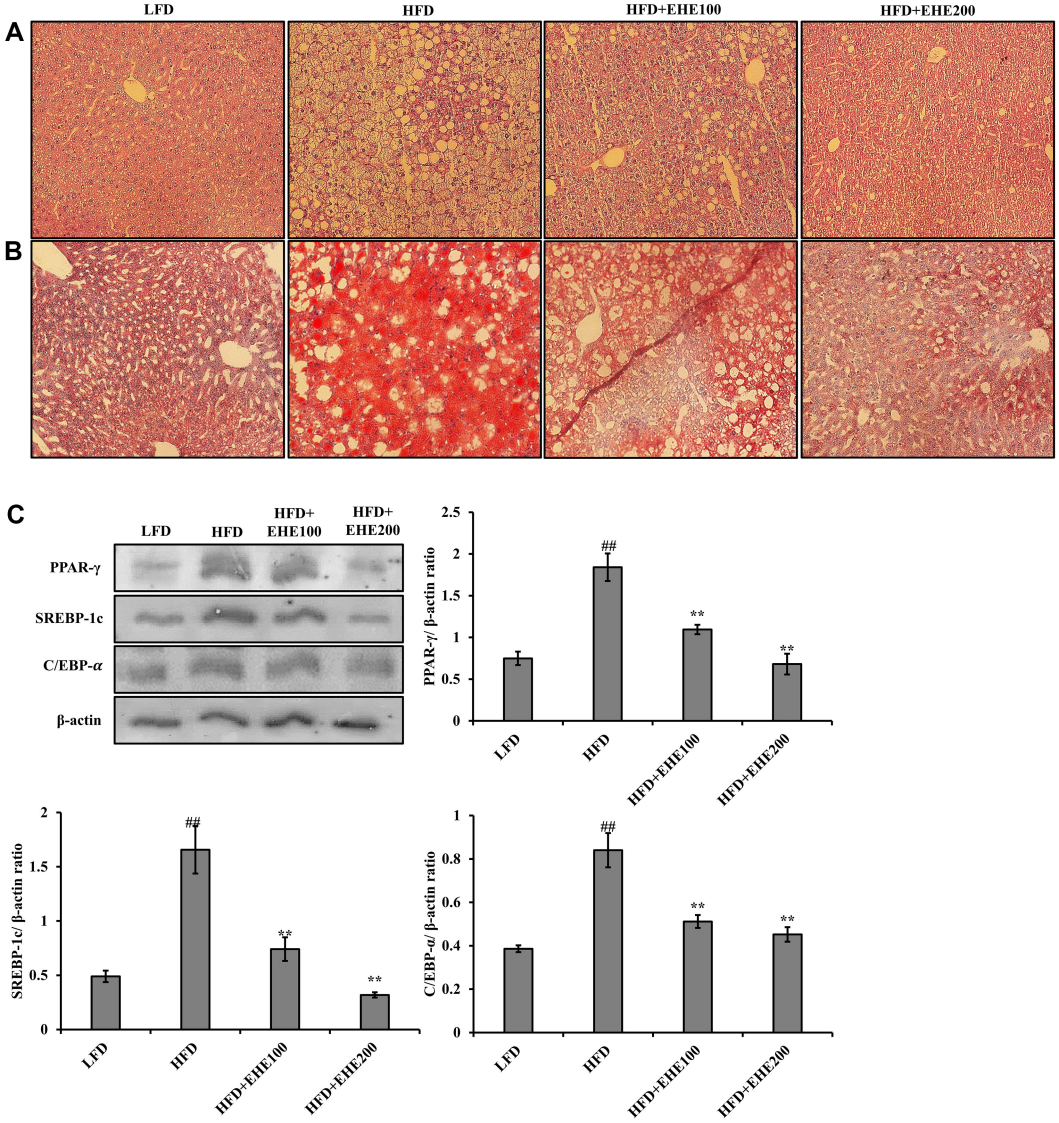
Effects of EHE on lipid accumulation and adipogenic transcription factors in liver tissue of HFD-fed mice. (**A**) Representative liver sections stained with H&E (200× magnification). (**B**) Representative liver sections stained with Oil Red O (200× magnification). (**C**) Western blot shows PPAR-γ, SREBP-1c, and C/EBP-α protein expression in liver tissues. β-actin served as the loading control. Relative protein expression levels, quantified as the band intensity relative to the intensity of the β-actin band, are shown for PPAR-γ, SREBP-1c, and C/EBP-α. All data are expressed as the mean ± SD of the experiment. ##*p* < 0.01 compared to the LFD group; **p < 0.01 compared to the HFD group.

To examine the effect of EHE on the liver, which controls lipid metabolism, H&E and Oil red O staining was performed in liver tissues derived from the four mouse groups (Fig. 7A and 7B). The H&E results confirmed that the liver tissues of the HFD group harbored larger and more lipid droplets compared to the liver tissues of the LFD group. These lipid droplets accumulated in the liver due to obesity. However, compared to the HFD group, less lipid accumulated in the livers of the EHE-treated groups, particularly the HFD + EHE200 group. The Oil red O staining results indicated that, in EHE-treated groups, the lipid areas (stained in red) were similar to the area observed in the LFD group. Thus, the histological results indicated that fat accumulated extensively in the livers of the HFD group, but with EHE treatment, fat accumulation was reduced. These findings suggested that EHE induced liver tissue regeneration and fat excretion.

### Effect of EHE on Adipogenic Transcription Factor

Western blot analyses were performed to evaluate the effects of EHE on the expression of adipogenic transcription factors in liver tissue (Fig. 7C). The results showed that PPAR-γ expression was significantly increased (*p* < 0.01) in the HFD group, compared to the LFD group, and significantly reduced (*p* < 0.01) in the HFD + EHE groups, compared to the HFD group. Similarly, both SREBP-1c and C/EBP-α expression levels were significantly increased (*p* < 0.01) in the HFD group compared to the LFD group, and significantly reduced (*p* < 0.01) in the HFD+EHE groups compared to the HFD group. These results suggested that that EHE suppressed the expression of adipogenic transcription factors, which in turn, inhibited the accumulation of fatty acids in liver cells and the differentiation of fat cells.

## Discussion

Recent studies have shown that obesity is both a cosmetic problem, due to the abnormal appearance, and an important health risk factor, due to increases in the risks of serious diseases, including type 2 diabetes, cardiovascular disease, stroke, and cancer. Unlike other medical fields, researchers in various fields conduct studies on obesity, because many complex factors are associated with obesity, such as exercise, nutrition, and psychosocial factors. Thus, ongoing research on obesity is focused on a variety of topics, such as health effects, energy balance regulation, management, and treatment [19].

Various studies focused on promoting the development of anti-obesity drugs are conducted around the world, but reports of side effects from currently marketed anti-obesity drugs have given rise to controversies, such as strengthening the dosing standards. Therefore, there is a demand for agents with excellent efficacy and high safety. In particular, much effort is spent on the discovery of natural raw materials with anti-obesity effects that are non-toxic and have no side effects [20, 21]. Previous studies demonstrated that EHE had no cytotoxicity and excellent activity in inhibiting adipocyte differentiation. Therefore, this study aimed to examine the potential of EHE as a novel anti-obesity agent and to identify its effects on anti-obesity-related factors.

The effect of DPPH radical scavenging activity was measured to evaluate the antioxidant activity of EHE. The DPPH assay measures the antioxidant effect within a short time. It is widely used to search for antioxidants in natural raw materials [22]. EHE showed excellent scavenging activity at a low concentration (25 μg/ml); its DPPH scavenging activity was about 56.32 ± 2.12% (Fig. 1).

HPLC was performed to identify and quantify quercetin, an active component in EHE. Quercetin was reported to have potential as a fat-lowering agent. It suggested mechanisms of action include positive effects on lipolysis, fatty acid intake, and adipogenesis inhibition [23-25]. According to various studies, quercetin has been shown to exert an anti-adipogenic effect in 3T3-L1 cells by inhibiting adipocyte-specific transcription factors, PPAR-γ and C/EBPα, and activating the AMPK signaling pathway [24, 25]. To evaluate the effects and the cytotoxicity of EHE and quercetin on 3T3-L1 adipocytes, Oil red O staining was performed in differentiated 3T3-L1 adipocytes treated with EHE or quercetin. The results showed that EHE significantly reduced the degree of fat accumulation (*p* < 0.001) compared to the control group (Fig. 3).

EHE prevented weight gain in C57BL/6J mice fed an HFD for 4 weeks. Compared to the LFD group, body weight significantly increased in the HFD group, but not with either dose of EHE (100 or 200 mg/kg), particularly with 200 mg/kg (*p* < 0.05; Fig. 4A). Similarly, the weights of abdominal and epididymal fat tissues (Fig. 4D and 4E) were lower in the EHE group compared to the HFD group. These results suggested that the weight loss effect was caused by a reduction in body fat.

Hyperlipidemia is an abnormal elevation in blood lipid concentrations, due to dysfunctional lipid metabolism. The presence of oxidized LDL-c increases white blood cell chemotaxis, and chemoattractant cytokines are produced in parietal cells, which lead to the formation of atherosclerosis in the blood vessel [26]. When treating hyperlipidemia, the primary goal is to lower LDL-c levels. In addition, it is important to increase HDL-c levels and lower TG levels. HDL-c independently removes excess lipids from tissues through the cholesterol reverse-transport process. Thus, HDL-c protects the endothelium and lowers the risk of coronary artery disease [26]. Our findings were consistent with those of Cho *et al*. [27], who reported that high HDL-c levels occurred in experimental animals fed an HFD due to an increase in the blood TC content induced by the HFD. However, our results also showed that the AIs in the HFD-EHE100 and HFD-EHE200 groups were significantly lower, at 0.37 ± 0.03 (*p* < 0.01) and 0.32 ± 0.03 (*p* < 0.001), respectively, compared to 0.52 ± 0.03 in the HFD group (Fig. 6E). Therefore, the EHE effects on lipid metabolism were examined by measuring serum lipid factors. The HFD-EHE group had significantly lower TG and LDL-c contents compared to the HFD group, but HDL-c levels were similar (Fig. 6A-6D). These results suggested that EHE improved lipid metabolism by selectively reducing TG and LDL-c levels, and maintaining the HDL-c content in the body.

Measuring the activity of liver enzymes released from the liver into the blood is one of the most useful methods for detecting liver damage. In particular, in the blood, AST and ALT enzymes transfer amino groups to keto acids and oxaloacetate. Thus, ALT catalyzes the transfer of an amino group from alanine to alpha-ketoglutarate to form pyruvate and glutamate [28]. The increased serum AST and ALT levels in the HFD group indicated that hepatic function was reduced. However, the HFD-EHE groups had lower serum AST and ALT levels compared to the HFD group; this suggested that EHE could restore liver function damaged by an HFD (Fig. 6F and 6G).

Lipogenesis and lipid metabolism occur mainly in the liver [29]. In mice, an HFD induces the expression of adipogenic transcription factors, PPAR-γ, SREBP, and C/EBP, in the liver [30]. PPAR-γ is a representative adipose-tissue differentiation transcription factor that plays an important role in body fat accumulation and adipocyte differentiation. SREBP is a transcription factor that plays an important role in liver fatty acid and cholesterol metabolism. C/EBP was first discovered among transcription factors related to adipocyte differentiation; it is expressed in both white and brown fat. It was shown that an increase in C/EBP expression led to an increase the expression of PPAR-γ [31]. We found that EHE suppressed the expression of PPAR-γ, SREBP-1c, and C/EBP-α (Fig. 7C). These effects may comprise the mechanism underlying the inhibitory effects of EHE on body fat accumulation and adipocyte differentiation.

This study demonstrated that EHE administration in HFD-induced obese mice inhibited gains in body, liver, and fat weights and prevented increases in serum TG, TC, HDL-c, LDL-c, AST, and ALT levels. In addition, our results suggested that EHE inhibited hepatic fat deposition associated with obesity by regulating genes related to adipocyte differentiation. These data indicated that quercetin-rich EHE may be an effective component for the treatment and prevention of HFD-induced obesity and lipid metabolism.

## Figures and Tables

**Table 1:** HPLC conditions for analyzing the quercetin content in EHE.

Parameter	Conditions		
Column	Agilent ZORBAX SB-C18 (4.6 mm × 250 mm, 5.0 μm, Agilent)		
Column temp.	Room temperature		
	Time (min)	A^1^ (%)	B^2^ (%)
	0	95	5
	5	75	25
Mobile phase (Gradient)	15	75	25
	25	50	50
	28	40	60
	33	30	70
	40	95	5
Detector	Shimadzu, SPD-M20A Diode Array Detector (334 nm)		
Flow rate	1.0 ml/min		
Injection volume	10 μl		
Run time	40 min		

^1^0.1% Phosphoric acid

^2^Acetonitrile

**Table 2:** Sequences of the primers used for real-time PCR.

Target	Primer sequences (5' to 3')	Accession No.
ACS1	GTCTTTGCCACATCCGACCTATC TTAGTGCAAACCCAGTTGTGCTTC	NM_007981.3
FAS	AGCACTGCCTTCGGTTCAGTC AAGAGCTGTGGAGGCCACTTG	NM_007988.3
FATP1	CAGACGGACGTGGCTGTGTA GCCGAGCATAGGATGCAAGAA	NM_011977.3
Perilipin	GATGAGAGCCATGACGACCAGA TGTGTACCACACCACCCAGGA	NM_175640.2
GAPDH	GTATGATCCACTCACGGCA GGTCTCGCTCCTGGAAGAGG	NM_002046.3
